# Delirium Post-Stroke—Influence on Post-Stroke Dementia (Research Study—Part of the PROPOLIS Study)

**DOI:** 10.3390/jcm9072165

**Published:** 2020-07-09

**Authors:** Jakub Droś, Katarzyna Kowalska, Paulina Pasińska, Aleksandra Szyper-Maciejowska, Agnieszka Gorzkowska, Aleksandra Klimkowicz-Mrowiec

**Affiliations:** 1 Doctoral School in Medical and Health Sciences, Jagiellonian University Medical College, 31-008 Kraków, Poland; jakub.dros@gmail.com; 2 Department of Neurology, Faculty of Medicine, Jagiellonian University Medical College, 31-008 Kraków, Poland; katarzyna.olga.kowalska@gmail.com (K.K.); pulinapotoczek@gmail.com (P.P.); 3 Department of Neurology, Jagiellonian University Hospital, 31-008 Kraków, Poland; ola.szyper@gmail.com; 4 Department of Neurorehabilitation, Faculty of Medical Sciences in Katowice, Medical University of Silesia, 40-055 Katowice, Poland; agorzkowska@sum.edu.pl

**Keywords:** stroke, post-stroke delirium, cognitive impairment, post-stroke dementia

## Abstract

Background: Previous research confirmed association between delirium and subsequent dementia in different clinical settings, but the impact of post-stroke delirium on cognitive functioning is still under-investigated. Therefore, we aimed to assess the risk of dementia among patients with stroke and in-hospital delirium. Methods: A total of 750 consecutive patients admitted to the stroke unit with acute stroke or transient ischemic attacks were screened for delirium, during the first seven days after admission. At the three- and twelve-month follow-up, patients underwent cognitive evaluation. The DSM-5 definition for dementia was used. Cases with pre-stroke dementia were excluded from the analysis. Results: Out of 691 included cases, 423 (61.22%) and 451 (65.27%) underwent cognitive evaluation, three and twelve months after stroke; 121 (28.61%) and 151 (33.48%) patients were diagnosed with dementia, respectively. Six (4.96%) patients with dementia, three months post-stroke did not meet the diagnostic criteria for dementia nine months later. After twelve months, 37 (24.50%) patients were diagnosed with dementia, first time after stroke. Delirium in hospital was an independent risk factor for dementia after three months (OR = 7.267, 95%CI 2.182–24.207, *p* = 0.001) but not twelve months after the stroke. Conclusions: Patients with stroke complicated by in-hospital delirium are at a higher risk for dementia at three but not twelve months post-stroke.

## 1. Introduction

Delirium, a transient condition of impaired attention and consciousness, is one of the most common complications in acute hospital admissions, leading to higher rates of post-discharge mortality and institutionalization [1]. According to the level of motor-activity disturbances, delirium is usually classified into four categories—hypoactive, hyperactive, mixed, or “no subtype”. Hypoactive delirium is found to have worse outcomes, as it is often missed and misinterpreted as fatigue, depression, or dementia, due to its less characteristic psychomotor presentation [2,3].

Occurrence of delirium complicating stroke in the acute phase varies between 6.7% to 66% [4]. There is an increasing interest in the effect of post-stroke delirium on healthcare outcomes, including mortality, disability, and neuropsychiatric status [5,6].

Although association between delirium in different clinical settings and subsequent cognitive impairment or dementia was confirmed in recent studies [7,8], the impact of in-hospital post-stroke delirium on cognitive functioning remains unclear. Post-stroke dementia affecting about one-third of stroke survivors [9], is a strong independent risk factor for early death, with a mortality rate up to 8.5 times higher than non-demented patients [10], a lower quality of life [11], and institutionalization [12]. There is some evidence in the literature that links delirium in the acute phase of stroke with subsequent decrease in cognitive functioning, however, the association is not sufficiently investigated [13].

Therefore, the aim of the present study was to investigate the influence of in-hospital post-stroke delirium on the incidence of dementia, in the short- and long-time perspective, after stroke.

## 2. Materials and Methods

This study was conducted as a part of a large single-center PRospective Observational POLIsh Study on post-stroke delirium (“PROPOLIS”) conducted at the Jagiellonian University Medical College in Kraków, Poland. This study investigated the prevalence of post-stroke delirium, its predictors, and the influence on short- and long-term prognosis [14]. All procedures performed in this study involving human participants were in accordance with the ethical standards of the Institutional and National Research Committee and with the 1964 Helsinki Declaration and its later amendments. Informed written consent was provided by each participant or a caregiver. The study protocol was approved by the Bioethics Committee of the Jagiellonian University (KBET/63/B/2014).

### 2.1. Study Design and Population

Consecutive patients admitted due to acute stroke or transient ischemic attack (TIA), to the Stroke Unit at the University Hospital in Kraków, who met the inclusion criteria for this study (patients >18 years of age, admitted within 48 h from the first stroke symptoms, Polish speakers), were investigated for the presence and the risk factors of delirium. Patients were screened for delirium every day from admission to the seventh day of hospitalization, with the abbreviated version of the Confusion Assessment Method (bCAM) or the Intensive Care Unit version (CAM-ICU), in patients with motor aphasia or those who could not communicate for other reasons [15,16]. To differentiate the delirium subtype, the Delirium Motor Checklist and Delirium Motor Subtype Scale 4 were applied [17,18].

Diagnosis of delirium was based on clinical observations and structural assessments. A resident neurologist, specially trained in delirium diagnosis, was responsible for screening for delirium, and a trained psychologist was responsible for cognitive assessment. The senior neurologist/neuropsychologist evaluated all data. The physicians rating the patients did not change during the study. Ward nurses completed a short questionnaire on patients’ behavior and cognitive fluctuations for each patient, in order to control for possible delirious symptoms during all 24 h. Delirium was diagnosed according to the criteria from the Diagnostic and Statistical Manual of Mental Disorders, Fifth Edition (DSM-5) [19].

### 2.2. Data Collection

Data was collected on socio-demographics factors (age, gender, education), medical history (comorbidities, medications, infections, biochemical disturbances, past medical history), and stroke-related (type and subtype of stroke, severity, stroke symptoms) variables. The Cumulative Illness Rating Scale (CIRS) was used as a general indicator of health status [20]. On admission, information was obtained from a spouse or caregiver regarding pre-stroke behavioral functioning on the Neuropsychiatric Inventory [21], and on the Polish version of the Informant Questionnaire on Cognitive Decline in the Elderly (IQCODE), in order to screen for pre-stroke dementia [22,23]. Patients with pre-stroke dementia, defined as the IQCODE, with a mean score of 4.0 or more [23,24], were excluded from further observation in this sub-study. Disability prior to admission was assessed by the modified Rankin Scale (mRS) [25].

All patients had neuroimaging (computed tomography/magnetic resonance) performed during admission. The severity of clinical deficit was graded by the National Institutes of Health Stroke Scale (NIHSS) [26], upon admission. Furthermore, the subtype of ischemic stroke was evaluated using the Trial of ORG 10,172 in the Acute Stroke Treatment (TOAST) classification [27]. Data regarding aphasia, neglect, or vision deficits were collected. Both cognitive and behavior/emotional functioning were also assessed. For cognition, the Montreal Cognitive Assessment (MoCA) [28] was performed twice; between the first and third day, and between the fourth and seventh day after stroke. For behavior/emotional function, patients were examined between the seventh and tenth day after stroke, using the Patient Health Questionnaire-9 (PHQ-9) [29], State-Trait Anxiety Inventory (STAI) [30], Buss-Durkee Hostility Inventory (BDHI) [31], and the Apathy Evaluation Scale (AES) [32] for evaluation of depression, anxiety, aggression, and apathy, respectively.

### 2.3. Follow-Up

All patients dismissed from the hospital were scheduled for a follow-up visit, three and twelve months after their stroke. If any patient did not attend a visit he/she was contacted by phone and a telephone interview was performed. In those cases where the patients could not be interviewed, a caregiver was contacted and interviewed. Information was collected on the current patient’s condition and medical history from the time of hospital discharge, including place of stay (home, rehabilitation hospital, long-term institution), occurrence, and type of recurrent stroke, or any other vascular event (heart attack, any heart surgery, cardioversion, endarterectomy), and current functional status (mRS) or death [33].

### 2.4. Outcome Assessment and Study Endpoints

At the three- and twelve-month follow-up visits in the outpatient clinic, the survivors underwent neuropsychological examination. In patients who refused full neuropsychological testing, only MoCA was performed [29]. Based on the meta-analysis of Shi et al., we used the following cut-off points of MoCA for dementia: ≤20 at the three-month follow-up and ≤23 at the twelve-month follow-up [34]. Patients who could not come to the clinic were assessed by a telephone version of MoCA (T-MoCA) [35]. Scores from the T-MoCA were proportionally rescaled, in relation to the maximal number of points to be obtained. To assess the cognitive status in patients who were not available for in-person and telephone interview due to severe impairments, or those who refused T-MoCA, the IQCODE was performed with a caregiver. Dementia was diagnosed if the mean score was ≥4.0 [23,24].

The final diagnosis of post-stroke dementia was based on the cognitive evaluation, functional assessment, and information provided by a caregiver. The DSM-5 diagnostic criteria for major neurocognitive disorder [19] were used.

The endpoints of this study were the prevalence of post-stroke dementia, three and twelve months after stroke, and the influence of in-hospital post-stroke delirium on dementia. The subgroup was patients after ischemic stroke.

### 2.5. Statistical Analysis

Statistical analysis was performed using the Statistica 13.3 software (StatSoft^®^, Kraków, Poland). The continuous values were presented as arithmetic means with standard deviations (SDs), or medians with interquartile ranges (IQRs), as appropriate. Qualitative variables were compared using the chi-squared test, with or without Yates’ correction, while the quantitative variables were compared with the Student’s *t*-test or Mann-Whitney U test, depending on normal or non-normal distribution, respectively. Considerable demographic and clinical factors, including delirium, were analyzed in univariate logistic regression models, to calculate their predictive values on post-stroke dementia at the follow-up time-points, presented as odds ratios (ORs) with a 95% confidence intervals (CIs). Then, delirium and other variables at *p*-value < 0.1 in the univariate analyses were included into the multivariate logistic regression models, as potential predictors, in search of independent risk factors, using the forward stepwise selection method. *p*-values < 0.05 were considered to be statistically significant.

## 3. Results

Out of the 750 patients included in the “PROPOLIS” study, 59 (7.87%) were excluded (45 with pre-stroke dementia and 14 due to lack of data on pre-stroke cognitive functioning). Among the remaining 691 patients, 598 (86.54%) had ischemic stroke, 47 (6.80%) had hemorrhagic stroke, and 46 (6.66%) had TIA. Delirium was identified in 174 (25.18%) subjects, of which the hyperactive subtype was observed in 27 cases (15.52%), hypoactive in 75 (43.10%), mixed in 62 (35.63%), and unspecified in 10 (5.75%) cases.

**Figure 1:**
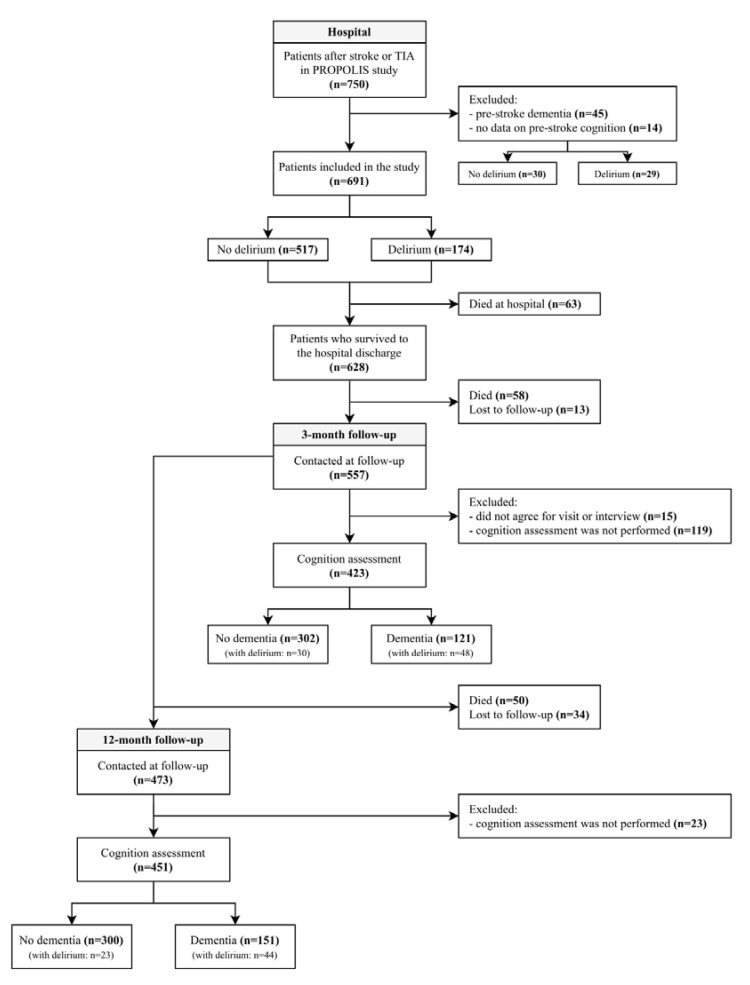
Study flowchart.

At the three-month follow-up, 557 of 691 (80.61%) patients were contacted, and cognitive evaluation was performed in 423 (61.22%) cases. At the twelve-month follow-up, 473 of 691 (68.45%) patients were contacted, and cognitive evaluation was performed in 451 (65.27%) cases. The study flowchart is presented in Figure 1.

### 3.1. Frequency of Dementia

Dementia was diagnosed in 121 of 423 (28.61%) and in 151 of 451 (33.48%) examined subjects, three and twelve months after stroke, respectively.

Out of 121 demented patients at the three-month follow-up, 78 (64.46%) were also diagnosed with dementia after twelve months of stroke, 6 (4.96%) had no dementia, 23 (19.01%) died, and 14 (11.57%) were not re-examined (11 cases were lost to follow-up and 3 patients did not agree for cognition assessment). After exclusion of the unexamined patients, the frequencies of dementia, no dementia, and death 12-months after stroke were observed in 78 (72.90%), 6 (5.61%), and 23 (21.50%) of 107 cases, respectively.

Out of the 151 demented patients at the twelve-month follow-up, 78 (51.66%) were also diagnosed with dementia three months after stroke, 37 (24.50%) had no previous dementia, and 36 (23.84%) were not examined at that time (5 patients did not agree for a visit or interview, 31 did not agree for cognition assessment). After excluding the unexamined patients, the frequencies of consecutive dementia and new cases of dementia were 78 (67.82%) and 37 (32.17%) of 115, respectively.

### 3.2. Three-Month Follow-Up

#### 3.2.1. Patients Characteristics

Patients with dementia had a higher level of disability and dependency (higher median mRS score, *p* < 0.001) and stayed longer in the hospital or institution (*p* = 0.006), but the groups did not differ in terms of frequency of recurrent stroke and cardiovascular events that occurred after hospital discharge (Table 1).

#### 3.2.2. Risk Factors for Dementia

Significant predictors of post-stroke dementia assessed three months after stroke in the univariate logistic regression analysis are presented in Table 2. Delirium in hospital occurred in 9.93% of non-demented and in 39.67% of demented subjects. Patients with delirium had a significantly higher risk of dementia (OR = 5.962, 95%CI 3.529–10.070, *p* < 0.001). No specific differences were observed regarding the subtypes of delirium.

In the multivariate logistic regression model, the lower MoCA scores assessed between the first and third day of hospital stay (OR = 0.822, 95%CI 0.761–0.888, *p* < 0.001) and the occurrence of delirium in hospital (OR = 7.267, 95%CI 2.182–24.207, *p* = 0.001) were independent risk factors for post-stroke dementia.

### 3.3. Twelve-Month Follow-Up

#### 3.3.1. Patient Characteristics

Patients with dementia had a higher level of disability and dependency (higher median mRS score, *p* < 0.001), stayed in the hospital or institution more often (*p* < 0.001), and experienced a recurrent stroke more often (*p* = 0.040), but the groups did not differ in terms of frequency of cardiovascular events that occurred after hospital discharge (Table 3).

#### 3.3.2. Risk Factors for Dementia

Significant predictors of post-stroke dementia, twelve months after stroke in the univariate logistic regression analysis are presented in Table 4. Delirium in hospital occurred in 7.67% and 29.14% of non-demented and demented subjects, respectively. Patients with delirium had a significantly higher risk of dementia (OR = 5.248, 95%CI 2.853–8.596, *p* < 0.001). No specific differences were observed regarding the subtypes of delirium.

In the multivariate logistic regression model, only a lower MoCA score assessed between the first and third day of hospital stay (OR = 0.789, 95%CI 0.728–0.856, *p* < 0.001) was an independent risk factor for post-stroke dementia.

### 3.4. Post-Hoc Sub-Analyses

In the first sub-analysis, the influence of delirium in the acute phase of stroke on the incidence of dementia twelve months after stroke was assessed; only patients with dementia diagnosed three and twelve months post-stroke were included into the “dementia” group (78 cases). Frequency of in-hospital delirium in this group was 37.18%, compared to 7.67% in the non-demented patients. Delirium was a significant risk factor in the univariate (OR 7.128, 95%CI 3.812–13.329, *p* < 0.001) but not in the multivariate logistic regression model.

The second sub-analysis assessed the influence of delirium in the acute phase of the stroke on the incidence of dementia twelve months after stroke; only patients with dementia diagnosed for the first time twelve months after stroke were included into “dementia” group (37 cases). In-hospital delirium in this group was diagnosed in 16.22% patients, compared to 7.67% in the non-demented patients. In both, univariate and multivariate logistic regression models, in-hospital delirium was not identified as a predictor of dementia.

## 4. Discussion

Results of our one-year prospective observational study showed that delirium affected cognition in the short-term after stroke but its impact declined with time.

Only few previous studies explored the association between post-stroke delirium and dementia, but these were difficult to compare because of the discrepancies in methodology. Their time of follow-up varied from one month to two years post-stroke. Furthermore, the authors defined cognitive impairment differently; i.e., as lower Mini–Mental State Examination score (Henon et al. [36], Sheng et al. [37], Dostovic et al. [38]), dementia according to DSM-III (Melkas et al. [39]), or dementia based on the results of the Clinical Dementia Rating scale and the Rotterdam-CAMCOG (van Rijsbergen et al. [40]). Additionally, these studies differed in the observation period for which the patients were screened for delirium after stroke. Finally, although cognitive impairment before stroke might independently affect the occurrence of both post-stroke delirium [41] and dementia [9], no author, except Henon et al., properly verified pre-stroke dementia as a confounder [13].

The results on the influence of in-hospital post-stroke delirium on dementia are summarized in the review of Ojagbemi and Ffytche [13]. These authors suggested a subsequent decrease in cognitive functioning within two years after stroke, among patients affected by delirium in the acute phase of stroke, however, the evidence level was estimated to be low.

A question exists regarding the most proper follow-up time, when cognition should be assessed after stroke, in order to deliver the most adequate data on its prevalence and association with stroke or delirium. No consensus was achieved as to how the rate of post-stroke dementia changes over the years, and if the clinical pattern of the early cognitive impairment after stroke was similar to that with delayed onset [42]. Between the three- and twelve-month follow-up time-points, we observed the increase in the prevalence of post-stroke dementia from 28.6% to 33.5%. For comparison, Caratozzolo et al. presented similar rates of 24.6% and 35.2%, three and twelve months after stroke, respectively [43], whereas Henon et al. showed a decrease from 22.8% at the six-month follow-up to 19.2% at the three-year follow-up [24].

Early assessment of cognitive status after stroke might lead to an overestimation of dementia among stroke survivors. The acute phase of stroke might cause rapid loss of some cognitive functions, which often improve during the following months of recovery [44,45]. In our study, 5% of patients with dementia diagnosed three months after stroke improved their cognitive functioning and could not be diagnosed with dementia nine months later.

On the other hand, stroke was associated with a faster decline in global cognition over years [46]. Altieri et al. found that 21.5% of patients who were non-demented three months after stroke, developed post-stroke dementia, during the four-year follow-up period [42]. In our study, almost one-fourth of patients diagnosed with dementia one year post-stroke, developed dementia between the third and twelfth month after stroke.

In our analysis, delirium was an independent risk factor for dementia diagnosed at the three-month follow-up, and this result was consistent with previous studies. No strong relation was observed twelve months after stroke, as delirium was only observed among the dependent predictors of dementia. We suspect that delirium and its psychological consequences could drive the cognitive decline that begins in the early post-stroke period, and improves within few months, whereas delayed onset of dementia might be associated with other mechanisms. Our observations are consistent with previous studies in other clinical settings, which separate delirium-related cognitive impairment from the pathological processes of classic dementia [7].

In-hospital delirium increased the risk of dementia three-months post-stroke, but it was not associated with dementia that developed for the first time between the third and twelfth month after stroke. The observations were in tune with the hypothesis that the etiology of post-stroke dementia developing in the early post-stroke period is different from dementia of delayed onset [42,47], and delirium might only influence early dementia. We believe these presumptions constitute the field for further investigation.

Our study was observational and based on the neuropsychological testing of post-stroke survivors. We did not perform any additional neuroimaging or examinations of potential biomarkers. To reveal the etiology of the association between post-stroke delirium and dementia, additional investigation for their molecular mechanism might be helpful. Recently, Cunningham et al. discovered the link between cerebrospinal fluid beta-amyloid and delirium incidence, after arthroplasty surgery, suggesting the potential impact of postoperative delirium on Alzheimer disease development [48]. Additionally, apolipoprotein e4 status should be considered, as it was shown to affect the risk of delirium [49] and post-stroke dementia [50]. These recent findings should encourage researchers to study the potential role of biomarkers in the diagnosis and outcome assessment for delirium and dementia in post-stroke population.

Avoiding or delaying some irreversible consequences of stroke might be achieved by preventing their predisposing factors. Thus, identifying post-stroke delirium as an independent predictor of dementia might be important in clinical practice, as dementia could be prevented using strategies targeted on delirium. Although there is no convincing evidence for the effectiveness of pharmacological prevention and treatment of delirium, especially in stroke survivors, some management is proven to be beneficial in reducing the delirium rate, such as reorientation, therapeutic activities, reduction of psychoactive drugs, early mobilization, promotion of sleep hygiene, maintenance of adequate hydration and nutrition, and provision of vision and hearing adaptations [41,51].

Our study also showed that worse cognitive status during the first three days after stroke (defined as the lower MoCA score) independently predicted dementia development in the short- and long-term perspective. Zietemann et al. and Wagle et al. found that cognitive assessment in the acute phase of the stroke predicted long-term cognitive functioning, and also the functional outcomes and mortality [52,53]. Thus, we suggest that cognitive evaluation during the first days following stroke, might be reasonable and could be helpful to plan rehabilitation and long-term care, however, further investigation of this aspect is still required.

In contrast to previous meta-analyses [9,54], except delirium and the lower MoCA score, we did not find any other strong significant predictors of post-stroke dementia, though many factors were associated with worse cognition at the three- and twelve- month follow-up in the univariate analysis. The role of the stroke location is among factors that still remains to be determined. Left hemisphere was significantly more often affected in patients with dementia, three and twelve months post stroke, but only in univariate analyses. Left hemispheric stroke was suggested to increase the risk of dementia in previous studies [9], but numerous studies did not find any relationship between stroke location and the risk of post-stroke dementia. Neuropsychological testing, for the most part, is based on verbal skills, which are mainly connected with the left hemisphere. Additionally, most patients are right-handed, therefore, the left hemisphere stroke impairs manual testing. In our study, handedness was not controlled, therefore, we could not draw stricter conclusions about this relationship. We also found that anticholinergic risk was a significant predictor of post-stroke dementia but only in univariate analysis. We might suspect that soon after stroke, the compensatory mechanisms might still be able to overcome the cholinergic deficit, but with time, when other processes join (i.e., degenerative ones), the decompensation is higher when patients are prone to anticholinergic drugs. However, we do not have enough evidence to support this assumption.

The advantages of our research include a large baseline study population, prospective design, and complex evaluation of the neuropsychiatric status in hospital. Furthermore, as diagnosis of delirium is often difficult and in many cases it might be missed, especially in stroke patients, due to prevalent language disorders, neglect, and mood disturbances, it can be mixed up with delirium or making proper assessment might be impossible, we assessed each patient systematically every day, using methods with high sensitivity and specificity, which were easy to administer. The same assessor administered the scale, making the bias of interobserver variation minimal. All patients were observed by the ward nurse for 24 h a day and the final diagnosis was complex, based on all regards.

The prevalence of dementia post-stroke was not a primary outcome in the “PROPOLIS” study, but the rate of dementia in this study was consistent with the results of the previous study from our center, where the assessment of dementia three months after stroke was the main outcome (28.6% vs. 31.4%, respectively) [23]. The methodology of both studies was very similar, except that they differed in terms of the diagnostic criteria for dementia (DSM-5 vs. DSM-IV) and the mean age of patients in both studies (69.4 years vs. 65.1 years).

Our study had several limitations. First, we included all adult patients of a wide age range, however, as the mean age of the cohort was similar to other studies, we expected that including young patients did not constitute a bias. Second, the incidence of post-stroke delirium in our sample could have been underestimated as the observation period was restricted to the first seven days. Third, most study participants were not under constant neurologic care in our center, after hospital discharge, so the study population could differ in the level of post-stroke rehabilitation plan, secondary prevention, and treatment of stroke complication. Fourth, the percentage of patients who did not agree for formal neuropsychological evaluation at the follow-up visits was relatively high.

## 5. Conclusions

Patients with delirium in the acute phase of stroke, irrespective of its subtype, were at higher risk for dementia, three months after stroke. There was no association between in-hospital delirium and dementia, twelve months after stroke.

Some dementia cases complicating stroke might reflect the dynamic, transient, and physiological consequences of delirium, which do not include permanent damage to the brain, whereas dementia developing as late post-stroke complication is probably associated with other mechanisms and factors. Further research, on a larger population and with a longer observational period, is required to determine the etiology of post-stroke dementia and to explain the differences between its early and delayed onset, after stroke.

## Figures and Tables

**Table 1:** Characteristics of the examined patients at the three-month follow-up.

Variable	Data	No Dementia	Dementia	*p*-Value
Number of patients	423	302/423 (71.39%)	121/423 (28.61%)	
Number of MoCA assessments *	423	221/302 (73.18%)	91/121 (75.21%)	
MoCA score **	312	26 (23–28)	16 (12–19)	<0.001
mRS **	413	1 (0–2)	3 (1–5)	<0.001
Place of stay				
- home *	417	217/297 (73.06%)	73/120 (60.83%)	0.006
- hospital *		72/297 (24.24%)	36/120 (30.00%)	
- institution *		7/297 (2.36%)	11/120 (9.17%)	
- others *		1/297 (0.34%)	0/120 (0%)	
Recurrent stroke *	416	1/296 (0.34%)	2/120 (1.67%)	0.417
Cardiovascular event *	415	5/296 (1.69%)	2/119 (1.68%)	0.678

* *n* (%); ** median (IQR); MoCA—Montreal Cognitive Assessment; mRS—Modified Rankin Scale.

**Table 2:** Predictors of post-stroke dementia at the three-month follow-up in the univariate logistic regression model.

Variable	Data	No Dementia	Dementia	OR	95%CI	*p*-Value
Male gender *	423	154/302 (50.99%)	48/121 (39.67%)	0.632	0.412-.0970	0.036
Age [years] **	423	66.5 (60–76)	78 (69–84)	1.069	1.046–1.092	<0.001
BMI [kg/m^2^] **	409	27.16 (23.99–30.22)	27.34 (23.88–30.12)	0.995	0.949–1.043	0.831
Higher education *	409	60/296 (20.27%)	10/113 (8.85%)	0.382	0.188–0.775	0.008
Length of education [years] **	399	12 (10–14)	10 (7–11)	0.788	0.723–0.860	<0.001
Hemorrhagic stroke *	423	17/302 (5.63%)	6/121 (4.96%)	0.875	0.336–2.274	0.784
TOAST classification						
- large-artery atherosclerosis *	365	29/256 (11.33%)	6/109 (5.50%)	0.456	0.184–1.132	0.091
- cardioembolism *	365	16/256 (6.25%)	5/109 (4.59%)	0.721	0.257–2.020	0.534
- small-vessel occlusion *	365	75/256 (29.30%)	51/109 (46.79%)	2.122	1.336–3.370	0.001
- other determined etiology *	365	132/256 (51.56%)	47/109 (43.12%)	0.712	0.453–1.118	0.141
- undetermined etiology *	365	4/256 (1.56%)	0/109 (0%)	-	-	-
Side of stroke						
- right hemisphere *	423	126/302 (41.72%)	42/121 (34.71%)	0.743	0.479–1.152	0.184
- left hemisphere *	423	133/302 (44.04%)	71/121 (58.68%)	1.804	1.177–2.766	0.007
- posterior part *	423	40/302 (13.25%)	6/121 (4.96%)	0.342	0.141–0.829	0.018
- more than one localization *	423	3/302 (0.99%)	2/121 (1.65%)	1.675	0.276–10.152	0.575
rt-Pa treatment *	423	70/302 (23.18%)	37/121 (30.58%)	1.460	0.912–2.336	0.115
Thrombectomy *	423	14/302 (4.64%)	7/121 (5.79%)	1.263	0.497–3.211	0.624
Medical history						
- hypertension *	423	215/302 (71.19%)	87/121 (71.90%)	1.035	0.648–1.654	0.884
- diabetes *	423	79/302 (26.16%)	40/121 (33.06%)	1.394	0.882–2.203	0.155
- atrial fibrillation *	423	47/302 (15.56%)	36/121 (29.75%)	2.298	1.396–3.784	0.001
- myocardial infraction *	423	44/302 (14.57%)	17/121 (14.05%)	0.958	0.524–1.754	0.891
- PCI or CABG *	423	31/302 (10.26%)	7/121 (5.79%)	0.537	0.230–1.254	0.151
- smoking – ever *	420	168/301 (55.81%)	43/119 (36.13%)	0.448	0.289–0.694	<0.001
- smoking – current *	420	97/301 (32.23%)	22/119 (18.49%)	0.477	0.283–0.804	0.006
- previous stroke or TIA *	421	39/300 (19.67%)	20/121 (16.53%)	0.809	0.463–1.413	0.456
CIRS, total score **	423	8 (5–11)	10 (6–13)	1.077	1.029–1.127	0.002
Medicines						
- antiplatelet drugs *	380	92/276 (33.33%)	42/104 (40.38%)	1.355	0.851–2.157	0.200
- anticoagulants *	380	36/276 (13.04%)	16/104 (15.38%)	1.212	0.641–2.293	0.554
- anticholinergic risk scale ***	380	0.07 ± 0.52	0.15 ± 0.75	1.237	0.874–1.751	0.229
- antidepressants *	349	8/245 (3.27%)	4/104 (3.85%)	1.185	0.349–4.205	1.185
- neuroleptics *	349	1/245 (0.41%)	0/104 (0%)	-	-	-
- benzodiazepines *	348	4/244 (1.64%)	2/104 (1.92%)	1.176	0.212–6.525	0.853
Pneumonia *	423	13/302 (4.30%)	21/121 (17.36%)	4.668	2.254–9.669	<0.001
Urinary tract infections *	423	73/294 (24.83%)	47/114 (41.23%)	2.124	1.344–3.355	0.001
Hospital stay [days] **	423	9 (8–10)	10 (9–13)	1.089	1.041–1.139	<0.001
Aphasia in hospital *	423	67/302 (22.19%)	50/121 (41.32%)	2.470	1.571–3.883	<0.001
Neglect in hospital *	423	29/302 (9.60%)	13/121 (10.74%)	1.133	0.568–2.262	0.723
Vision deficits in hospital *	423	74/302 (24.50%)	55/121 (45.45%)	2.568	1.648–4.001	<0.001
NIHSS at admission **	423	4 (2–9)	7 (4–14)	1.085	1.049–1.122	<0.001
Pre-hospital mRS **	423	0 (0–0)	0 (0–1)	1.584	1.243–2.018	<0.001
mRS at 7–10 day **	423	1 (0–3)	3 (1–4)	1.485	1.308–1.686	<0.001
Laboratory tests at admission						
- WBC [×10^3^/μL] **	309	7.9 (6.5–9.5)	8.0 (6.7–10.1)	1.023	0.943–1.109	0.585
- glucose [mmol/L] **	375	6.7 (5.6–8.1)	6.4 (5.5–8.9)	1.010	0.940–1.085	0.787
- Na^+^ [mmol/L] **	379	139.0 (137.0–141.0)	139.0 (137.0–141.0)	0.985	0.932–1.041	0.600
- K^+^ [mmol/L] **	380	3.9 (3.6–4.2)	4.0 (3.7–4.4)	1.434	0.977–2.105	0.065
- creatinine [μmol/L] **	378	80.0 (66.0–96.0)	78.5 (63.0–99.5)	1.002	0.998–1.006	0.265
CRP in hospital [mg/L] **	412	3.8 (1.7–12.0)	8.4 (3.4–23.4)	1.005	1.001–1.010	0.013
NPI, total score **^/^***	349	0 (0–8)/6.1 ± 11.6	3 (0–12)/7.3 ± 10.7	1.009	0.990–1.028	0.373
- agitation/aggression ***	349	2.1 ± 5.3	1.6 ± 4.7	0.978	0.930–1.029	0.400
- mood ***	349	2.4 ± 4.9	1.8 ± 3.2	0.968	0.914–1.025	0.263
- frontal ***	349	1.9 ± 4.5	1.8 ± 3.3	0.994	0.941–1.051	0.846
- psychosis ***	349	0.3 ± 1.8	0.4 ± 1.8	1.040	0.923–1.172	0.518
Cognition assessment						
- MoCA at 1–3-day, score **	341	23 (18.5–25)	15 (12–19)	0.821	0.778–0.865	<0.001
- MoCA at 4–7-day, score **	338	24 (21–27)	17 (12.5–20)	0.816	0.775–0.859	<0.001
PHQ-9 at 7–10-day, score **	363	4 (2–9)	7 (2–11.5)	1.067	1.019–1.118	0.006
AES at 7–10-day, score **	329	29 (21–38)	38.5 (29–45.5)	1.061	1.036–1.086	<0.001
STAI-S at 7–10-day, score **	360	26 (28–45)	39 (30.5–48)	1.013	0.993–1.033	0.204
STAI-T at 7–10-day, score **	360	41 (32–47.5)	44 (36–50)	1.026	1.002–1.050	0.030
BDHI at 7–10-day, total score **	339	48 (36–68)	55 (40–76)	1.014	1.003–1.026	0.014
- assault **	339	6 (2–8)	8 (4–10)	1.096	1.031–1.166	0.004
- indirect hostility **	339	6 (4–10)	6 (4–10)	0.971	0.909–1.037	0.379
- irritability **	339	6 (4–10)	8 (6–10)	1.060	1.000–1.123	0.050
- negativism **	339	4 (2–6)	4 (2–8)	1.049	0.971–1.132	0.225
- resentment **	339	5 (2–8)	8 (4–12)	1.113	1.049–1.181	<0.001
- suspicion **	339	10 (6–14)	12 (8–14)	1.046	0.995–1.100	0.079
- verbal hostility **	339	12 (8–16)	12 (8–18)	1.042	0.995–1.090	0.080
- guilt **	339	8 (6–12)	12 (6–14)	1.056	1.006–1.109	0.027
Delirium in hospital *	423	30/302 (9.93%)	48/121 (39.67%)	5.962	3.529–10.070	<0.001
Delirium type						
- hyperactive *	423	5/302 (1.66%)	11/121 (9.09%)	5.940	2.018–17.483	0.001
- hypoactive *	423	12/302 (3.97%)	19/121 (14.88%)	4.223	1.967–9.069	<0.001
- mixed *	423	11/302 (3.64%)	15/121 (12.40%)	3.744	1.667–8.408	0.001
Delirium length [days] **	78	3 (1–7)	3 (1.5–6)	0.965	0.796–1.170	0.717

* *n* (%); ** median (IQR); *** mean ± SD; BMI—body mass index; TOAST—Trial of Org 10,172 in Acute Stroke Treatment; rt-Pa—recombinant tissue plasminogen activator; PCI—percutaneous coronary interventions; CABG—coronary artery bypass graft; TIA—transient ischemic attack; CIRS—Cumulative Illness Rating Scale; NIHSS—National Institutes of Health Stroke Scale; mRS—Modified Rankin Scale; WBC—white blood cells; CRP—C-reactive protein; NPI—Neuropsychiatric Inventory; MoCA—Montreal Cognitive Assessment; PHQ-9—Patient Health Questionnaire-9; STAI—State-Trait Anxiety Inventory (S-state, T-trait); BDHI—Buss-Durkee Hostility Inventory; AES—Apathy Evaluation Scale.

**Table 3:** Characteristics of the examined patients at the twelve-month follow-up.

Variable	Data	No Dementia	Dementia	*p*-Value
Number of patients	451	300/451 (66.52%)	151/451 (33.48%)	
Number of MoCA assessments *	451	197/300 (65.67%)	113/151 (74.83%)	
MoCA, score **	310	26 (25–28)	18 (14–21)	<0.001
mRS **	451	1 (0–2)	2 (1–4)	<0.001
Place of stay				
- home *	440	233/290 (80.34%)	92/150 (61.33%)	<0.001
- hospital *		25/290 (8.62%)	14/150 (9.33%)	
- institution *		29/290 (10.00%)	32/150 (21.33%)	
- others *		3/290 (1.03%)	12/150 (8.00%)	
Recurrent stroke *	448	8/298 (2.68%)	11/150 (7.33%)	0.040
Cardiovascular event *	448	8/298 (2.68%)	6/150 (4.00%)	0.640

* *n* (%); ** median (IQR); MoCA—Montreal Cognitive Assessment; mRS—Modified Rankin Scale.

**Table 4:** Predictors of post-stroke dementia at the twelve-month follow-up in the univariate logistic regression model.

Variable	Data	No Dementia	Dementia	OR	95%CI	*p*-Value
Male gender *	451	159/300 (53.00%)	68/151 (45.03%)	0.727	0.491–1.076	0.111
Age [years] **	451	65 (58–75)	74 (67–82)	1.063	1.043–1.083	<0.001
BMI [kg/m^2^] **	443	26.64 (23.66–30.48)	25.95 (23.88–29.38)	0.981	0.941–1.022	0.355
Higher education *	439	71/295 (24/07%)	16/144 (11.11%)	0.394	0.220–0.707	0.002
Length of education [years] **	432	12 (10–14.5)	10 (8–12)	0.805	0.747–0.867	<0.001
Hemorrhagic stroke *	451	12/300 (4.00%)	10/151 (6.62%)	1.702	0.718–4.035	0.227
TOAST classification						
- large-artery atherosclerosis *	391	26/262 (9.92%)	12/129 (9.30%)	0.931	0.454–1.911	0.845
- cardioembolism *	391	15/262 (5.73%)	6/129 (4.65%)	0.803	0.304–2.121	0.658
- small-vessel occlusion *	391	73/262 (27.86%)	49/129 (37.98%)	1.586	1.015–2.478	0.043
- other determined etiology *	391	144/262 (54.96%)	62/129 (48.06%)	0.758	0.497–1.157	0.199
- undetermined etiology *	391	4/262 (1.53%)	0/129 (0%)	-	-	-
Side of stroke						
- right hemisphere *	451	124/300 (41.33%)	59/151 (39.07%)	0.910	0.610–1.357	0.645
- left hemisphere *	451	127/300 (42.33%)	79/151 (52.32%)	1.495	1.009–2.214	0.045
- posterior part *	451	45/300 (15.00%)	10/151 (6.62%)	0.402	0.197–0.822	0.013
- more than one localization *	451	4/300 (1.33%)	3/151 (1.99%)	1.500	0.331–6.790	0.599
rt-Pa treatment *	451	72/300 (24.00%)	38/151 (25.17%)	1.065	0.677–1.675	0.786
Thrombectomy *	451	15/300 (5.00%)	10/151 (6.62%)	1.348	0.590–3.076	0.479
Medical history						
- hypertension *	451	202/300 (67.33%)	113/151 (74.83%)	1.443	0.929–2.239	0.102
- diabetes *	451	68/300 (22.67%)	49/151 (32.45%)	1.639	1.061–2.532	0.026
- atrial fibrillation *	451	43/300 (14.33%)	39/151 (25.83%)	2.081	1.279–3.387	0.003
- myocardial infraction *	451	39/300 (13.00%)	22/151 (14.57%)	1.141	0.650–2.005	0.646
- PCI or CABG *	451	25/300 (8.33%)	14/151 (9.27%)	1.124	0.566–2.231	0.738
- smoking – ever *	448	163/299 (54.52%)	64/149 (42.95%)	0.628	0.423–0.934	0.022
- smoking – current *	448	95/299 (31.77%)	25/149 (16.78%)	0.433	0.264–0.709	<0.001
- previous stroke or TIA *	449	49/299 (16.39%)	32/150 (21.33%)	1.384	0.842–2.273	0.200
CIRS, total score **	451	7 (4–10)	10 (6–13)	1.120	1.072–1.171	<0.001
Medicines						
- antiplatelet drugs *	410	73/276 (26.45%)	51/134 (38.06%)	1.709	1.101–2.652	0.017
- anticoagulants *	410	33/276 (11.96%)	22/134 (16.42%)	1.446	0.807–2.594	0.216
- anticholinergic risk scale ***	410	0.02 ± 0.22	0.15 ± 0.74	2.000	1.058–3.781	0.033
- antidepressants *	376	4/247 (1.62%)	5/129 (3.88%)	2.450	0.646–9.285	0.188
- neuroleptics *	376	1/247 (0.40%)	2/129 (1.55%)	3.874	0.348–43.135	0.271
- benzodiazepines *	375	4/246 (1.63%)	2/129 (1.55%)	0.953	0.172–5.273	0.956
Pneumonia *	451	11/300 (3.67%)	17/151 (11.26%)	3.333	1.519–7.312	0.003
Urinary tract infections *	451	61/291 (20.96%)	55/146 (37.67%)	2.279	1.471–3.531	<0.001
Hospital stay [days] **	451	9 (8–10)	10 (9–12)	1.085	1.033–1.139	0.001
Aphasia in hospital *	451	63/300 (21.00%)	52/151 (34.44%)	1.976	1.278–3.055	0.002
Neglect in hospital *	451	27/300 (9.00%)	18/151 (11.92%)	1.368	0.728–2.573	0.330
Vision deficits in hospital *	451	66/300 (22.00%)	54/151 (35.76%)	1.974	1.283–3.036	0.002
NIHSS at admission **	451	3 (1–7)	6 (3–12)	1.077	1.041–1.113	<0.001
Pre-hospital mRS **	451	0 (0–0)	0 (0–1)	1.582	1.226–2.042	<0.001
mRS at 7–10 day **	451	1 (0–2)	2 (1–4)	1.500	1.319–1.705	<0.001
Laboratory tests at admission						
- WBC [×10^3^/μL] **	329	7.7 (6.3–9.4)	7.8 (6.7–9.3)	1.028	0.944–1.118	0.529
- glucose [mmol/L] **	403	6.6 (5.6–8.1)	6.9 (5.5–9.0)	1.063	0.995–1.134	0.068
- Na^+^ [mmol/L] **	408	139.0 (138.0–141.0)	139.0 (136.0–141.0)	0.967	0.925–1.012	0.148
- K^+^ [mmol/L] **	409	3.9 (3.6–4.2)	4.0 (3.7–4.3)	1.466	1.004–2.139	0.047
- creatinine [μmol/L] **	406	79.0 (66.0–92.0)	79.0 (66.0–97.5)	1.003	0.997–1.009	0.303
CRP in hospital [mg/L] **	440	3.7 (1.6–10.9)	6.6 (2.2–17.0)	1.009	1.002–1.015	0.009
NPI, total score **^/^***	376	0 (0–6)/4.5 ± 9.9	3 (0–11)/7.5 ± 10.9	1.027	1.006–1.049	0.012
- agitation/aggression ***	376	1.8 ± 5.3	1.8 ± 4.2	1.001	0.959–1.045	0.961
- mood ***	376	1.8 ± 4.1	2.2 ± 4.1	1.021	0.971–1.074	0.421
- frontal ***	376	1.4 ± 3.7	1.8 ± 3.4	1.032	0.975–1.092	0.282
- psychosis ***	376	0.2 ± 1.4	0.7 ± 3.0	1.102	0.987–1.231	0.085
Cognition assessment						
- MoCA at 1–3-day, score **	377	24 (20–26)	18 (14–21)	0.818	0.778–0.860	<0.001
- MoCA at 4–7-day, score **	365	25 (22–27)	19 (12–23)	0.819	0.779–0.861	<0.001
PHQ-9 at 7–10-day, score **	399	4 (1–8)	6 (2–10)	1.055	1.012–1.100	0.012
AES at 7–10-day, score **	364	27 (21–36)	33 (24–44)	1.046	1.024–1.068	<0.001
STAI-S at 7–10-day, score **	395	35 (28–45)	38 (30–49)	1.015	0.996–1.033	0.119
STAI-T at 7–10-day, score **	395	40 (32–47)	42 (35–50)	1.021	1.000–1.043	0.046
BDHI at 7–10-day, total score **	371	48 (36–66)	54 (40–70)	1.011	1.000–1.022	0.045
- assault **	371	6 (2–8)	6 (4–10)	1.081	1.023–1.143	0.006
- indirect hostility **	371	6 (4–10)	8 (4–10)	1.010	0.952–1.071	0.750
- irritability **	371	6 (4–10)	8 (4–10)	1.018	0.965–1.074	0.515
- negativism **	371	4 (2–6)	4 (2–8)	1.010	0.941–1.084	0.780
- resentment **	371	4 (2–8)	6 (2–10)	1.081	1.023–1.143	0.006
- suspicion **	371	10 (6–14)	12 (6–14)	1.039	0.993–1.087	0.102
- verbal hostility **	371	12 (8–16)	12 (8–18)	1.028	0.986–1.071	0.195
- guilt **	371	8 (6–12)	10 (6–14)	1.040	0.994–1.087	0.089
Delirium in hospital *	451	23/300 (7.67%)	44/151 (29.14%)	4.952	2.853–8.596	<0.001
Delirium type						
- hyperactive *	451	4/300 (1.33%)	10/151 (6.62%)	5.248	1.618–17.025	0.006
- hypoactive *	451	9/300 (3.00%)	16/151 (10.60%)	3.832	1.651–8.892	0.002
- mixed *	451	7/300 (2.33%)	15/151 (9.27%)	4.277	1.688–10.838	0.002
Delirium length [days] **	67	2 (1–6)	3 (2–7)	1.155	0.934–1.430	0.184

* *n* (%); ** median (IQR); *** mean ± SD; BMI—body mass index; TOAST—Trial of Org 10,172 in Acute Stroke Treatment; rt-Pa—recombinant tissue plasminogen activator; PCI—percutaneous coronary interventions; CABG—coronary artery bypass graft; TIA—transient ischemic attack; CIRS—Cumulative Illness Rating Scale; NIHSS—National Institutes of Health Stroke Scale; mRS—Modified Rankin Scale; WBC—white blood cells; CRP—C-reactive protein; NPI—Neuropsychiatric Inventory; MoCA—Montreal Cognitive Assessment; PHQ-9—Patient Health Questionnaire-9; STAI—State-Trait Anxiety Inventory (S-state, T-trait); BDHI—Buss-Durkee Hostility Inventory; AES—Apathy Evaluation Scale.
